# Phone-based audience response system as an adjunct in orthodontic teaching of undergraduate dental students: a cross-over randomised controlled trial

**DOI:** 10.1186/s12909-020-02363-3

**Published:** 2020-11-16

**Authors:** Fahad Alharbi, Khulud F. Alazmi, Bashar R. El Momani, Lubna Al-Muzian, Mark Wertheimer, Anas Almukhtar, Mohammed Almuzian

**Affiliations:** 1 Department of Preventive Dental Sciences / College of Dentistry, Prince Sattam Bin Abdulaziz University, Al-Kharj, 11942 Saudi Arabia; 2 Ministry of Health, Kingdom of Saudi Arabia, Riyadh, Saudi Arabia; 3 Queen Alia Military Hospital, Dental Corps-Orthodontics department, Royal Medical Services of Jordan Armed Forces, Amman, Jordan; 4 Glasgow Orthodontic Academy, Edinburgh, UK; 5 Private Orthodontic Practice, Johannesburg, South Africa; 6 College of Dentistry, University of Mosul, Mosul, Iraq; 7 Australia & Honorary Research Fellow, University of Glasgow, Glasgow, Scotland; 8 Orthodontist (Private clinic, UK) and Honorary Research Fellow, Edinburgh Dental Institute, University of Edinburgh, Edinburgh, UK

**Keywords:** Teaching methods, Medical education, Corrective orthodontics, Curriculum, Dental education, Dental research, Educational technology methods, Dental students

## Abstract

**Background:**

The advent of electronic teaching facilities improves tutor-student communication. This study aims to explore the effectiveness of Phone-Based Audience Response System (PB-ARS), as an adjunctive pedagogy tool to enhance the retention of orthodontic information by dental students; and to explore the students’ perception of PB-ARS.

**Methods:**

This cross-over clustered randomised control trial included 34 males who were in the final year of their undergraduate dental training. Participants were allocated to one of two event groups (G1 and G2) using computer-generated randomisation. Both groups simultaneously attended two different traditional lectures (L 1 and L2) a week apart. During L1, PB-ARS was used as an adjunct to conventional presentation to teach G1 participants, (PB-ARS group) while G2’s participants acted as a control group (CG), and were taught using a traditional presentation. In the second week (L2), the interventions were crossed-over. Participants from both groups completed pre- and post-lecture multiple-choice questionnaires (MCQ) to assess their short-term retention of information. Their performance in the final MCQ exam (10 weeks following L2) was tracked to assess the long-term retention of the information. Participants also completed post-lecture questionnaires to evaluate their perceptions.

**Results:**

Twenty-nine and 31 participants from the CG and PB-ARS group completed this trial, respectively. Although 87.5% of students in the PB-ARS group showed an improvement in their immediate post-lecture scores compared with 79.3% for the CG, it was statistically insignificant (*p* = 0.465). Similarly, the intervention showed an insignificant effect on the long-term retention of the knowledge (*p* = 0.560).

There was a mildly but favourable attitude of students towards the use of PB-ARS. However, the difference in the overall level of satisfaction between both groups was statistically insignificant (*p* = 0.183).

**Conclusion:**

PB-ARS has a minimal and insignificant effect on the short- and long-term retention of orthodontic knowledge by male undergraduate dental students. PB-ARS was the preferred adjunct tool to conventional classroom teaching. Due to the limitations of this trial, a long-term randomised controlled trial with a larger sample size is recommended.

**Supplementary Information:**

The online version contains supplementary material available at 10.1186/s12909-020-02363-3.

## Background

Undergraduate teaching has always been regarded as the core foundation in the development and maturation of undergraduate students. Lecturing and learning are synonymous; hence, the underlying principles governing the appropriate teaching approaches are mainly aimed at encouraging student-tutor communication, enabling consistent feedback, and establishing an interactive teaching model [1, 2] . Other crucial pedagogical elements in any given course are to promote deep learning, though most post-secondary educational courses rely on traditional teacher-centered and passive student participation approaches [3]. Evidence shows that didactic lectures require a high level of motivation and concentration, yet yield low retention of knowledge, this being approximately 5–50% of the taught subject [4]. On the other hand, interactive adult teaching enhances deep, self-directed and reflective learning. Similarly, active question-based education stimulates and enhances learning more than didactic lectures [5].

An audience response system (ARS) is an electronic device introduced to be of value in teaching and education in parallel, as it transforms traditional didactic lectures into a more interactive learning process [6]. An ARS was first used for education in 1991 by Rice University to teach statistics [7]. The implementation of electronic wireless interactive technology has gradually stepped into the educational paradigm, acting as a novel pathway towards a more developed student-learning process, thus promoting self-directed learning. ARS, also called personal response systems or clickers, act as interactive tools allowing students to share their knowledge instantly, by voting electronically on an on-screen or on-handset set of questions.

The ARS can be hard-wired or wireless. Wireless ARS can either be a specific handset-based ARS connected via radio or internet to a master handset controller, or personal smartphone-based ARS using web poll, short message service (SMS) or direct wireless connection through smartphone applications. Most ARS allow the running of multiple-choice questions [8]. However, the recently introduced ARS provide an additional option of running open-ended and dichotomous questions using either text or multimedia-based (pictures or video) questions.

Recently developed ARS allow instant evaluation of students’ responses against their peers to confirm whether there is a need for further elaboration of the primary vital points, thus, assisting tutors to redesign the delivery of learning materials [9, 10]. Furthermore, students’ responses can be sent anonymously. This allows the students to answer in a non-threatening environment, eliminating the main barrier of active participation, which is embarrassment [11]. An example of the ARS is the Poll Everywhere which is a smartphone application that has a feature enabling the administrator to launch open-ended and dichotomous questions, using either text or multimedia-based (pictures) materials, and then collects and analyses the answers from the users (students) instantly.

A trial based in England showed that ARS could have a positive influence on students’ concentration levels, resulting in a subsequent improvement in the retention of orthodontic knowledge when implemented in small group seminars [8]. Another study showed that ARS promote interactions during orthodontic lectures, but with little effect on short-term information recall [12]. The same research also showed that students preferred using the ARS while attending lectures and seminars, since they found it easier to understand, interact, and later, participate [12]. As a result, it is expected that the retention of knowledge would be better than traditional teaching methods. An additional application of the phone-based ARS (PB-ARS) in the current COVID-19 pandemic, is the versatility in engaging students during distant online teaching, building interactivity in the virtual classroom, and possibly compensating for the lack of face-face interaction. Studies concluded that the use of ARS in virtual teaching during the COVID-19 era, effectively improved the delivery of the teaching material and enhanced the interactivity of the learners [13–15]. Another group of researchers reported similar findings when they used PB-ARS in teaching chemistry during the COVID-19 period. Although those reports at best represent expert opinions which lack the robustness of other research design, they pave the way for the potential use of PB-ARS in remote teaching [16].

On the other hand, there are generic obstacles in the uses of ARS, such as subscription rate, maintenance, training, troubleshooting and technical support. Also, it seems that there is conflicting evidence regarding the effectiveness of ARS. Robson and colleagues found that the benefits of the ARS are insignificant; nevertheless, there was a small improvement in knowledge gained by the ARS group compared with the control group [12]. Hence, it is essential to reach a consensus regarding the effectiveness of ARS in orthodontic pedagogy.

The aim of this cross-over randomised controlled trial (RCT) was (1) to investigate the effectiveness of PB-ARS using text- and multimedia-based questions on the retention of information by male undergraduate dental trainees; (2) to explore the students’ perception and acceptance of PB-ARS. The null hypothesis stated that there is no difference in terms of knowledge retention and students’ perceptions when PB-ARS is used as an adjunct to didactic orthodontic teaching of undergraduate dental students.

## Methods

### Funding, ethical considerations and registration

This trial was self-funded and approved by the local committee of research at the College of Dentistry at Prince Sattam Bin Abdulaziz University (PSAU) (1439–03-001). The trial was registered with ClinicalTrials.gov Protocol Registration and Results System (NCT04336813), however, the protocol was not published. The authors declare that there is no financial interest or conflict of interest in this trial.

### Study design

The trial was designed as a cross-over clustered randomised control trial (each group was a cluster).

### Setting and consent

The study was commenced at the College of Dentistry, PSAU in Alkharj City in Saudi Arabia. Written consent was obtained from all participants before starting the trial.

### Participants

The eligibility criteria included undergraduate students in the fourth year of their dental training with no prior orthodontic education. Students who were registered in the course for the second time were excluded to lessen the bias associated with increased knowledge. The cohort of the trial involved 34 undergraduate dental students.

### Randomisation

Participants were allocated to one of the two even groups using computer-generated randomisation. Participants in the control group (CG) were taught through the conventional model using PowerPoint presentation. Participants in the intervention arm used Phone-Based Audience Response System (PB-ARS group) as an adjunct during the PowerPoint presentation. The sequence of random allocation was concealed from the researchers who recruited the participants. Each group consisted of 17 male students.

### Intervention

#### Lectures

Simultaneously, CG and PB-ARS groups attended two lectures, the first lecture (L1) titled “Management of Class III Malocclusion” while the second lecture (L2) was titled “Management of Open Bite and Cross-bite”. L1 and L2 were delivered at the main campus of PSAU College of Dentistry. L1 and L2 were delivered identically in all aspects, including:

- the presentation platform (PowerPoint, Microsoft Corp, Redmond, WA),
- the lecturer (both L1 and L2 were given by the same registered specialist orthodontist (F.A.), and
- the duration of the lectures which was 60 min.

Learning outcomes of the delivered lectures were based on learning objectives and outcomes as specified by the National Commission for Academic Accreditation and Assessment in Saudi Arabia.

Before L1, students were instructed to register with the PB-ARS and to download its application (Poll Everywhere, San Francisco, California, USA, https://www.polleverywhere.com). Extra smartphones were accessible to students who did not have smartphones during the lectures. Students were blinded from their allocations until the beginning of L1.

Before the L1 and L2, both groups completed a validated multiple-choice question (MCQs) formative assessment. During L1, the participants in the PB-ARS group had access to an interactive poll of new questions regarding the taught topic, via their smartphones. The participants in the PB-ARS group were allowed to read the questions and answer them. Participants in the CG were blinded from those questions. To assess the improvement in the students’ performance, at the end of L1, both groups again completed the pre-lecture MCQs test. A similar protocol was undertaken during L2 a week later, except that the groups were crossed-over. Hence, the group which had PB-ARS integrated during L1 were blinded from the poll of questions during L2, and vice versa. At the end of L1 and L2, participants in the PB-ARS and CG groups answered a set of questions regarding their experience with the lecture.

### Formative MCQs exam

MCQ formative tests consisted of 20 questions related to the topics taught during L1 and L2. The maximum achievable score was 20. To reduce the carry-over effect, the PB-ARS questions during the lectures were different from the MCQ formative written exam sheet. Two authors piloted the bank of questions to ensure its content validity and reliability. Content validity was tested using test matrix and expert judgment. The reliability test was estimated using inter-rater reliability. A correlation of more than 0.7 was considered acceptable.

### Summative exam

Both groups attended their final written summative exams 10 weeks after L2. The final exam was in MCQ format. The summative written exams covered questions from all dental and medical subjects taught during the second semester in the fourth year of undergraduate dental training at the College of Dentistry, PSAU. The summative written exam included five randomly selected questions relevant to the orthodontic subjects taught in L1 and L2. The written exam questions were identical for all students and delivered under controlled exam conditions. The summative written exam scores specific to L1 and L2 questions were traced and collected using an Excel spreadsheet by an independent tutor to reduce reporting bias. The maximum achievable score for the five questions relevant to the subjects taught in L1 and L2 was 5.

### Students’ perception

At the end of L1 and L2, participants of the CG completed a set of questionnaires regarding their experience with the lecture. Similarly, participants of PB-ARS group completed another set of questionnaires (add1). The questionnaire of the CG consisted of 9 questions that assessed understanding of the topic of the lecture, possibility of participation in the lecture, interaction with the tutor, and total level of satisfaction. The questionnaire of the intervention group (PB-ARS) included an additional 4 questions specific to PB-ARS that aimed to assess the perception of using PB-ARS as an adjunct to conventional teaching. To enhance the validity and reliability of the questionnaire, the original English version of the questionnaires, adopted from a previous study with close similarity to our assessed cohorts, was also used in our study [8]. Additionally, the questionnaire was distributed between the authors to reduce ambiguity and modify confusing questions, if deemed necessary. Each question was answered using a 0–10 scale. The response of the students was categorised into five categories. The 5 categories of responses were: strongly disagree (score 0–1), disagree (score 2–4), neutral (score 5), agree (score 6–8) and strongly agree (score 9–10).

### Analysis of the results

Students’ responses and scores were exported into an Excel spreadsheet. Students who failed to attend the summative exam and decided to take the “resit” exam were excluded from this trial to reduce the effect of time as a confounding factor. An intention to treat [17] analysis was adopted to deal with dropouts and missing data of non-compliant participants. Data was analysed by a blinded statistician using SPSS 22. Pre- and post-lecture formative assessment scores were analysed and compared using cross-over analysis with the Mann–Whitney U test, while a t-test was used to analyse summative exam scores.

## Results

The entire cohort of fourth-year undergraduate dental students (34 students, aged 23.27 years ±0.86) were enrolled in this cross-over randomised trial. During L1, three students were absent (one from the PB-ARS group and two from the CG). During L2, the trial’s arms were crossed over, and three students were absent, all from the CG. In L1, only 31 students attended, 16 students were allocated to the PB-ARS group while the rest were allocated to the CG. In L2, 31 students attended the lecture, 17 students were allocated to the PB-ARS group and 14 students were allocated to the CG. On average, the percentage of students’ attendance of the L1 and L2 were similar.

**Fig. 1:**
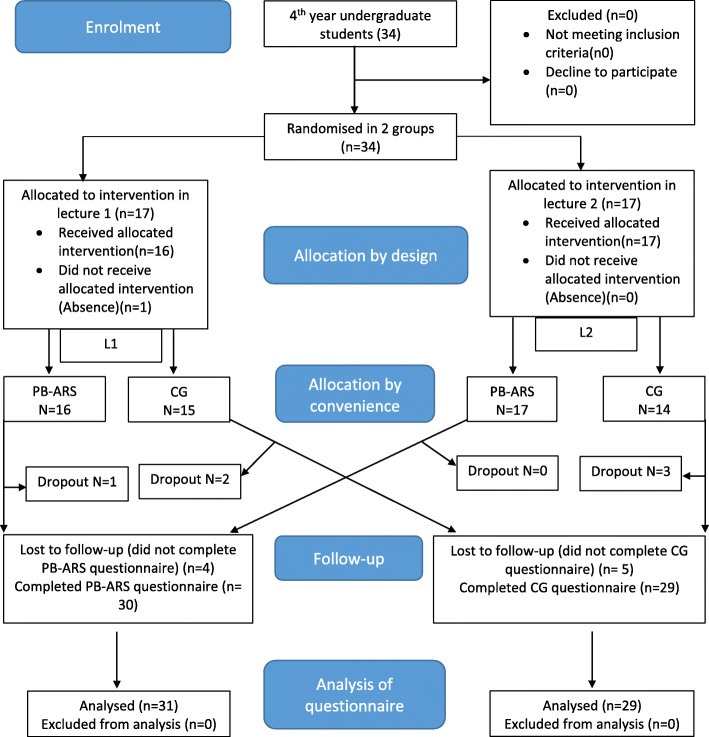
CONSORT Flow Diagram of the trial

In total after L1 and L2, 33 students from the PB-ARS group and 29 students from the CG completed the formative exam. Thirty students from the PB-ARS group and 29 students from the CG completed the perception’s questionnaire. Figure 1 shows the CONSORT flow diagram of the study.

### Questionnaires

The results of both questionnaires are displayed in Tables 1 and 2. In both groups, the majority of the participants agreed that the lectures were understandable (86.6% for PB-ARS group; 83% for CG) and felt that they enjoyed the presented topics (83.3% for PB-ARS group; 83% for CG). Students in both groups felt they were capable of participating in the active discussion during the lectures (83.3% for PB-ARS group; 83% for CG), and agreed that they were more receptive to questioning during the lectures, in particular when PB-ARS was implemented (76.7%% for PB-ARS group compared to 72% for CG). Participants of the PB-ARS group reported a higher level of concentration compared with those in the CG, 80 and 66% respectively.

**Table 1:** Students responses in the PB-ARS

Question		Response				
		Strongly agree	Agree	Indifferent	Disagree	Strongly disagree
1	Do you feel that you understood the topic that was being delivered today?	9 (30%)	17 (56.6%)	2 (6.7%)	2 (6.7%)	0 (0%)
2	How interesting did you find the seminar?	10 (33.3%)	14 (46.7%)	2 (6.7%)	4 (13.3%)	0 (0%)
3	Did you enjoy the seminar today?	13 (43.3%)	12 (40%)	0 (0%)	4 (13.3%)	1 (3.3%)
4	Do you find it easy to concentrate?	9 (30%)	15 (50%)	4 (13.3%)	2 (6.7%)	0 (0%)
5	Did you find it easy to participate in the session?	9 (30%)	16 (53.3%)	1 (3.3%)	4 (13.3%)	0 (0%)
6	Was there an opportunity to ask questions?	9 (30%)	14 (46.7%)	3 (10%)	2 (6.7%)	2 (6.7%)
7	Do you feel you were able to give feedback to your tutor?	7 (23.3%)	18 (60%)	3 (10%)	2 (6.7%)	0 (0%)
8	Did you prepare for this seminar?	1 (3.3%)	7 (23.3%)	3 (10%)	13 (43.3%)	6 (20%)
9	Were you more likely to answer questions using the Polleverywhere?	9 (30%)	15 (50%)	3 (10%)	3 (10%)	1 (3.3%)
10	Do you prefer the conventional method of seminar teaching?	11 (36.7%)	10 (33.3%)	1 (3.3%)	4 (13.3%)	4 (13.3%)
11	Do you prefer the Polleverywhere?	9 (30%)	14 (46.7%)	1 (3.3%)	4 (13.3%)	2 (6.7%)
12	Will you be more likely to prepare for the next seminar if you know that Polleverywhere will be used?	9 (30%)	16 (53.3%)	1 (3.3%)	2 (6.7%)	2 (6.7%)
13	Overall, rate your level of satisfaction with the seminar?	8 (26.7%)	19 (63.3%)	1 (3.3%)	2 (6.7%)	0 (0%)

**Table 2:** Students responses in the CG

Question	Response				
	Strongly agree	Agree	Indifferent	Disagree	Strongly disagree
Do you feel that you understood the topic that was being delivered today?	6 (21%)	18 (62%)	1 (3%)	4 (14%)	0 (0%)
How interesting did you find the seminar?	7 (24%)	17 (59%)	2 (7%)	3 (10%)	0 (0%)
Did you enjoy the seminar today?	9 (31%)	14 (48%)	2 (7%)	4 (14%)	0 (0%)
Do you find it easy to concentrate?	6 (21%)	13 (45%)	3 (10%)	6 (21%)	1 (3%)
Did you find it easy to participate in the session?	7 (24%)	17 (59%)	4 (14%)	0 (0%)	1 (3%)
Was there an opportunity to ask questions?	7 (24%)	14 (48%)	3 (10%)	5 (17%)	0 (0%)
Do you feel you were able to give feedback to your tutor?	3 (10%)	19 (66%)	4 (14%)	3 (10%)	0 (0%)
Did you prepare for this seminar?	3 (10%)	12 (41%)	1 (3%)	7 (24%)	6 (21%)
Overall, rate your level of satisfaction with the seminar?	4 (14%)	20 (69%)	3 (10%)	2 (7%)	0 (0%)

The majority of participants (70%) were satisfied and preferred (76.7%) the use of PB-ARS (Poll Everywhere mobile app) during the lecture. Participants reported that they were more likely to prepare for future lectures if PB-ARS was to be used (83.3%). In terms of the overall satisfaction levels, there was a statistically insignificant difference between the two groups (90% for PB-ARS group; 83% for CG; median and mode questionnaire score for both groups = 4, *p* = 0.183).

### Retention of knowledge

Analysis of the students’ performance during the formative exam was carried out to assess short-term recall of knowledge. Twenty-nine students from the CG arm and 31 students from the intervention arm (PB-ARS group) undertook the pre-lecture and post-lecture MCQ formative assessments. 87.5% of students in the PB-ARS group showed improvement in their scores after the intervention, compared with 79.3% in the CG. The means of the pre-lecture exam score (the maximum achievable score was 20) for the CG and the PB-ARS groups were (6.4, SD 3) and (7.2, SD 2.25) respectively. The mean of the post-lecture exam score (the maximum achievable score was 20) was (8.89, SD 3.86) for the CG and (10, SD 2.74) for the PB-ARS group. Nevertheless, there was no statistically significant difference between the groups (mean difference (MD) = 2.63, confidence interval 1.74–3.52, *p* = 0.465).

Analysis of the students’ performance during the summative exam was carried out to assess long-term retention of knowledge. The mean for the summative exam score (the maximum achievable score was 5) was (2.87, SD1.51) for the CG and (3.06, SD 1.49) for the PB-ARS group; the mean difference was statistically insignificant (MD 0.194, 95% CI (− 0.467) -0.854, *p* = 0.560).

## Discussion

Recently, several reports have explored the effectiveness of ARS in medical teaching and education. A recent meta-analysis found that the use of ARS technology in learning had positive effects on both cognitive and non-cognitive learning outcomes [18]. However, few of the conducted trials have studied ARS application for undergraduate dental teaching programs, in particular in the field of orthodontics [19, 20]. The material of the discipline of orthodontics is considered to have a relatively high level of speciality, and consequently is considered to be out of the scope of practice of general dentists. Therefore, it has always been a dilemma for dental faculties and tutors to effectively measure their students’ essential understanding of orthodontic knowledge and principles [21].

Orthodontics as a taught subject at PSAU starts in the fourth year of the Bachelor of Dental Science (B.D.S.) degree. Thus, the choice was made to select this cohort of students who had no previous exposure to orthodontic material. The entire class was included in the study. This ensured no selection bias since all the assessed participants had the same level of dental knowledge.

One tutor (F.A.) delivered both taught lectures (L1, L2), 1 week apart, and the groups were then crossed-over. This further reduced bias and enhanced the blinded cross-over protocol. Both groups had a formative written exam following each lecture, to assess the short-term retention of information, and a final summative written exam, 10 weeks later, to evaluate the long-term recall of the knowledge.

The null hypothesis of our trial was accepted. The results showed that the majority of the participants in both groups rated the lectures as enjoyable, interesting, and found it easy to understand the taught topic. This finding was in line with those reported in the literature [5, 8, 12, 17, 22]. Besides these findings, the participants in the intervention group reported higher levels of concentration, probably due to the need for active participation during the lecture. Students responded positively towards upcoming lectures in which PB-ARS was planned to be used. This might represent the effectiveness of PB-ARS in building interest in the subject material, though the difference in the overall rating was statistically insignificant. This was in agreement with previous studies [12]. A study has shown that PB-ARS provides the students with a safe teaching environment due to the anonymity of the users [23]. Other studies have reported that the use of clickers in the classroom improved students’ attention during the lectures [24, 25]. Nevertheless, a previous study conducted in 2006 found that while the implementation of ARS might encounter difficulty in lecture preparation, the ARS fostered student participation [26].

Still, it is crucial to note that the use of smartphones as PB-ARS might have some disadvantages which could limit the feasibility within the classroom. For instance, smartphones might create a source of student distraction and demand internet access. In addition, faculty members might need further training on the use of this fast-developing modern technology. To lower the probability for biased results, participation in this study was not compulsory. No students were compelled to complete the questionnaire and participants were allowed to leave the lecture hall at their will.

In our trial, the formative and summative exam scores of the participants from the PB-ARS group improved marginally compared with those of the control group. This is different from previously reported findings [27, 28]. Stowell and Nelson reported a noticeable, but statistically insignificant improvement in student learning curves [29]. Moreover, students’ attendance in our trial was almost identical in both arms of the study, unlike the previous study [30]. This could be because students involved in this study had been informed that they were participating in a research project, which might have increased the level of interest and alertness during both lectures (Hawthorne effect).

### Limitations of the study

Although the sample included all undergraduate students in their fourth year of dental training, retrospective sample size calculations showed that our study was underpowered. Robson and colleagues have suggested conducting a parallel multi-centre research study, including several dental schools, to increase the sample size to include a minimum of 74 students per group [12]. Nevertheless, undesirable effects such as lack of standardisation of lecture delivery would then need to be accounted for.

Another point to consider when interpreting the results of our study is the “carry-over effect”, as the effect of the intervention may not have dissipated when crossing over the groups. This might have contaminated students’ experiences, and had an effect on their responses, despite the fact that the taught topics were different in the presented lectures. A further long-term parallel-arm randomised controlled trial with a larger sample size is required to evaluate the effectiveness of using PB-ARS in teaching orthodontics to dental undergraduate students.

In addition, in our trial, it was not possible to include female undergraduate students since the trial was undertaken at a gender-specific dental school. That might have prevented the evaluation of gender as a variable while analysing students’ attitudes toward PB-ARS [31]. Previous studies have reported insignificant differences among both genders [31–33].

## Conclusions

The PB-ARS with text- and multimedia-based questions has no significant effect on the short- and long-term retention of orthodontic knowledge by the undergraduate dental students. However, PB-ARS is perceived positively by students, and it might be a useful adjunctive interactive educational tool. A further long-term parallel-arm randomised controlled trial with a larger sample size is required.

## Supplementary Information

**Additional file 1:Appendix 1** Questionnaire for students.

## Data Availability

Data and material are available at the orthodontic department in the College of Dentistry, Prince Sattam Bin Abdulaziz University/Alkharj/ Saudi Arabia.

## Abbreviations

ARS: Audience response system/s
SMS: Short message service
RCT: Randomised controlled trial
PB-ARS: Phone-based ARS
MCQs: Multiple-choice questions
L1: Lecture 1
L2: Lecture 2
