# Supplementary material for: Phone-based audience response system as an adjunct in orthodontic teaching of undergraduate dental students: a cross-over randomised controlled trial
Source: BMC Med Educ. 2020 Nov 16;20:435. doi: 10.1186/s12909-020-02363-3 (PMC7668011; doi:10.1186/s12909-020-02363-3)
Supplement: Supplementary file 1 — Additional file 1:Appendix 1 Questionnaire for students. [file 12909_2020_2363_MOESM1_ESM.docx]

**Appendix 1 Questionnaire for students**

**Questionnaire of the control group**

*Please circle a number on the scale from 0–10, where 0 corresponds with no/not at all and 10 corresponds with yes/very much*

1. Do you feel that you understood the topic that was being delivered today?

*0 1 2 3 4 5 6 7 8 9 10*

2. How interesting did you find the seminar?

*0 1 2 3 4 5 6 7 8 9 10*

3. Did you enjoy the seminar today?

*0 1 2 3 4 5 6 7 8 9 10*

4. Do you find it easy to concentrate?

*0 1 2 3 4 5 6 7 8 9 10*

5. Did you find it easy to participate in the session?

*0 1 2 3 4 5 6 7 8 9 10*

6. Was there an opportunity to ask questions?

*0 1 2 3 4 5 6 7 8 9 10*

7. Do you feel you were able to give feedback to your tutor?

*0 1 2 3 4 5 6 7 8 9 10*

8. Did you prepare for this seminar?

*0 1 2 3 4 5 6 7 8 9 10*

9. Overall, rate your level of satisfaction with the seminar

*0 1 2 3 4 5 6 7 8 9 10*

Are there any comments you would like to make about the seminar? (Please continue overleaf if necessary.) -----------------------------------------------------------------------

**Questionnaire of the intervention (PB-ARS) group**

*Please circle a number on the scale from 0–10, where 0 corresponds with no/not at all, and 10 corresponds with yes/very much*

1. Do you feel that you understood the topic that was being delivered today?

*0 1 2 3 4 5 6 7 8 9 10*

2. How interesting did you find the seminar?

*0 1 2 3 4 5 6 7 8 9 10*

3. Did you enjoy the seminar today?

*0 1 2 3 4 5 6 7 8 9 10*

4. Do you find it easy to concentrate?

*0 1 2 3 4 5 6 7 8 9 10*

5. Did you find it easy to participate in the session?

*0 1 2 3 4 5 6 7 8 9 10*

6. Was there an opportunity to ask questions?

*0 1 2 3 4 5 6 7 8 9 10*

7. Do you feel you were able to give feedback to your tutor?

*0 1 2 3 4 5 6 7 8 9 10*

8. Did you prepare for this seminar?

*0 1 2 3 4 5 6 7 8 9 10*

9. Were you more likely to answer questions using the PollEverywhere ?

*0 1 2 3 4 5 6 7 8 9 10*

10. Do you prefer the conventional method of seminar teaching?

*0 1 2 3 4 5 6 7 8 9 10*

11. Do you prefer the PollEverywhere?

*0 1 2 3 4 5 6 7 8 9 10*

12. Will you be more likely to prepare for the next seminar if you know that ARS will be used?

*0 1 2 3 4 5 6 7 8 9 10*

13. Overall, rate your level of satisfaction with the seminar?

*0 1 2 3 4 5 6 7 8 9 10*

Are there any comments you would like to make about the seminar and the ARS? (Please continue overleaf if necessary.) -----------------------------------------------------------------------
